# Comparing the dosimetric characteristics of the electron beam from dedicated intraoperative and conventional radiotherapy accelerators

**DOI:** 10.1120/jacmp.v16i2.5017

**Published:** 2015-03-08

**Authors:** Hamid Reza Baghani, Seyed Mahmoud Reza Aghamiri, Seyed Rabi Mahdavi, Mohammad Esmail Akbari, Hamid Reza Mirzaei

**Affiliations:** ^1^ Radiation Medicine Department Shahid Beheshti University Tehran; ^2^ Medical Physics Department Iran University of Medical Science Tehran; ^3^ Cancer Research Center, Shahid Beheshti University of Medical Science Tehran Iran

**Keywords:** electron beam, dosimetric characteristics, IORT, conventional radiotherapy

## Abstract

The specific design of the mobile dedicated intraoperative radiotherapy (IORT) accelerators and different electron beam collimation system can change the dosimetric characteristics of electron beam with respect to the conventional accelerators. The aim of this study is to measure and compare the dosimetric characteristics of electron beam produced by intraoperative and conventional radiotherapy accelerators. To this end, percentage depth dose along clinical axis (PDD), transverse dose profile (TDP), and output factor of LIAC IORT and Varian 2100C/D conventional radiotherapy accelerators were measured and compared. TDPs were recorded at depth of maximum dose. The results of this work showed that depths of maximum dose, $R_{90},R_{50}$, and $R_{P}$ for LIAC beam are lower than those of Varian beam. Furthermore, for all energies, surface doses related to the LIAC beam are substantially higher than those of Varian beam. The symmetry and flatness of LIAC beam profiles are more desirable compared to the Varian ones. Contrary to Varian accelerator, output factor of LIAC beam substantially increases with a decrease in the size of the applicator. Dosimetric characteristics of beveled IORT applicators along clinical axis were different from those of the flat ones. From these results, it can be concluded that dosimetric characteristics of intraoperative electron beam are substantially different from those of conventional clinical electron beam. The dosimetric characteristics of the LIAC electron beam make it a useful tool for intraoperative radiotherapy purposes.

PACS number: 87.56.‐v, 87.56.bd

## I. INTRODUCTION

Intraoperative radiation therapy (IORT) is designed to deliver a high single fraction of radiation dose to the patient after surgery and under anesthesia.^1^, ^2^, ^3^ There are three different methods to perform this kind of radiotherapy: IOERT, HDR‐IORT, and Low KV IORT,^4^, ^5^ of which, IOERT is the most popular, because of the desired dose distribution,^(6)^ limited penetration of the electron beam, and treatment time. In recent years, various kinds of mobile dedicated accelerators like Mobetron, NOVAC7, and LIAC are introduced for IOERT.^5^, ^7^, ^8^, ^9^ These accelerators are small, light, and portable ones that are only capable of producing electrons with the energies up to 12 MeV (in order to minimize the neutron production^(10)^). Dedicated IORT accelerators have a very high dose per pulse electron beam^(11)^ which strongly reduces the irradiation time needed to achieve the prescribed dose. On the other hand, calibration of the electron beam produced by such types of accelerators is not a simple task using ion chambers. The presence of high dose per pulse electron beam causes some degree of uncertainty in determination of recombination correction factor of ions formed in the chamber sensitive volume.^12^, ^13^, ^14^ This uncertainty is due to the fact that the free electron fraction created in the chamber sensitive volume is not taken into account during determination of recombination correction factor^(15)^ using the two voltage analysis (TVA) method presented by the IAEA TRS‐398 and AAPM TG‐51 protocols.^16^, ^17^ Therefore, the extended Boag theory and Laitano or Di Martino formalism^18^, ^19^ must be applied to determine the recombination correction factor of ion chambers used for calibration of intraoperative radiotherapy electron beams. The most important differences between these mobile dedicated and conventional accelerators are the electron beam collimation system and the type of the applicator used with each of them. The most commonly utilized applicators with these mobile accelerators are the flat and beveled base cylindrical tubes of various diameters. Two types of collimation systems are employed to collimate the electron beam produced by these mobile radiotherapy machines, namely hard and soft docking system.^5^, ^11^ In the first one, the IORT applicator is connected to the machine head, while in the second one, the applicator is fixed to the surgical bed as well as being in contact with the patient, while there is no connection to the accelerator.^20^, ^21^ Using this type of applicators and collimation systems can change the physical characteristics (energy, spatial, and angular distribution) of the electron beam. Therefore, it can be expected that the dosimetric properties of the electron beam produced by these mobile accelerators are different from those of conventional accelerators. The aim of this study is to measure and compare the dosimetric characteristics of the electron beam from IORT and conventional radiotherapy accelerators. To this end, percentage depth dose along clinical axis (PDD), transverse dose profile (TDP), and output factor related to the LIAC (12 MeV model) mobile dedicated accelerator (Sordina SpA, Vicenza, Italy) and the Varian 2100C/D conventional accelerator (Varian Medical Systems, Palo Alto, CA) were measured and compared.

## II. MATERIALS AND METHODS

### A. LIAC (12 MeV Model) mobile dedicated accelerator

LIAC is a mobile dedicated accelerator for radiotherapy in operating room. There are two types of LIAC: 10 MeV and 12 MeV, with nominal energies of 4, 6, 8, and 10 MeV and 6, 8, 10, and 12 MeV, respectively.^(22)^ The 12 MeV model was employed in this study.

Dose rate of this machine can be adjusted from 5 to 30 Gy per min using pulse repetition frequency (PRF) from 1 to 60 Hz. Length of accelerating structure, including 19 self‐focusing cavities, is equal to 92.5 cm. A 55 μm titanium foil is located at the exit of accelerating structure. Scattering foil consists of an aluminum sheet with 820 μm thickness to meet the following conditions: broadening the pencil electron beam incidences on the titanium exit window, reducing the probability of the neutron production at high energies,^4^, ^23^, ^24^ controlling the X‐ray contamination, and limiting the length of employed applicator. Beam monitoring system consists of two monitor ion chambers, each one includes two 5 μm aluminum electrodes. A 100 μm Mylar sheet is placed at the exit of the beam monitoring system.

The employed applicators are made of sterillizable PMMA cylindrical tubes with 5 mm thickness and 60 cm length. Diameter and base angle of these applicators change between 3 to 10 (3, 4, 5, 6, 7, 8, and 10) cm and 0° to 45° (0°, 15°, 30°, and 45°), respectively. Although the length of these applicators is 60 cm, the distance between the scattering foil and the end of the applicator and, hence, the SSD is equal to 71.3 cm.^(23)^ The hard docking mechanism is applied for electron beam collimation.^(4)^

### B. Measurements

#### B.1 Percentage depth dose (PDD)

To measure the PDDs related to the flat base cylindrical IORT applicators and square conventional applicators, an Advanced Markus chamber (TM 34045; PTW, Freiburg, Germany) was employed. Advanced Markus is a vented parallel plate chamber with the sensitive volume of 0.02 cm^3^. Chamber was connected to a TANDEM digital electrometer (PTW) and the operating voltage was set to 300 volts. All of the measurements were performed inside an automatic MP3 water phantom tank (MP3‐XS; PTW) according to the recommendations of the IAEA TRS‐398 protocol.^(16)^ Chamber positions inside the phantom were automatically controlled using a TBA control unit (PTW). To obtain the PDD at different energies, at first percentage depth ionization (PDI) curve was measured, and then, this curve was converted to PDD using the water‐to‐air stopping power ratios according to the IAEA TRS‐398 protocol.^(16)^ Finally, depth of maximum dose $\left(R_{100}\right)$, depth of 90% dose $\left(R_{90}\right)$, depth of 50% dose $\left(R_{50}\right)$, practical range ($R_{p}$), depth at which the tangent to linear portion of PDD curve intersects the extrapolated bremsstrahlung background, surface dose $\left(\%D_{s}\right)$, and X‐ray background absorbed dose^(25)^ (in terms of maximum dose percentage) of the electron beams from both machines at different energies were extracted from depth dose curves using PTW MEPHYSTO beam analysis software.

The major problem concerning our PDD measurements was related the surface dose. The Advanced Markus chamber has a 1.3 mm thick entrance window (including waterproof cap, entrance foil, and air gap between the cap and the entrance foil) which make it impossible to directly measure the surface dose. Therefore, the surface dose $\left(\%D_{s}\right)$ was obtained through applying the cubic spline^(26)^ extrapolation method to the measured data at depths beyond 2 mm (taking into account the 1 mm water‐equivalent window thickness of ion chamber).

The PDDs of beveled IORT applicators were measured with a PinPoint chamber (TM 31014; PTW). This chamber is a waterproof cylindrical detector with 1 mm radius and sensitive volume of 0.015 cm^3^ which has no dependency to beam incidence angle. PDDs were obtained along clinical axis, in this case clinical axis is perpendicular to the surface and intersects geometrical axis at the field center, inside the MP3 water phantom and chamber voltage was set to 400 volts during scans. It should be mentioned that the same procedure for flat applicators was also followed to obtain the surface dose of beveled ones.

The measurements related to the LIAC accelerator, were carried out at SSD=71.3 cm, and field diameter of 10 cm (cylindrical applicator) for energies of 6, 8, 10, and 12 MeV, and in the case of the Varian accelerator, at SSD of 100 cm, and field size of $10\times 10\;{\text{cm}}^{2}$ (standard square applicator), for energies of 6, 9, and 12 MeV. In order to suppress the effects of accelerator output variations, a Semiflex chamber (TM 31010, PTW) was used as a reference detector^(26)^ during scans.

#### B.2 Transverse dose profile (TDP)

In order to measure the transverse dose profile at above‐mentioned energies, the effective point of measurement of Advanced Markus chamber, in chamber center on the entrance foil, was placed at the $d_{\text{max}}$ related to each electron beam energy (taking into account the 1 mm water‐equivalent window thickness of ion chamber) inside the automatic MP3 water phantom and TDP was obtained in cross plane (according to recommendations of AAPM TG‐106 protocol^(26)^) using the mentioned equipments (TANDEM electrometer and TBA control unit). Then, the left penumbra, right penumbra, flatness, and symmetry of radiation field were extracted from TDPs of both machines using PTW MEPHYSTO beam analysis software. LIAC measurements were performed using 10 cm flat base cylindrical applicator at SSD of 71.3 cm, and Varian ones were carried out at 100 cm SSD and square field size of $10\times 10\;{\text{cm}}^{2}$. The Semiflex chamber was also used as the reference detector.

#### B.3 Output factor

Output factor (Eq. (1)) is defined as the ratio of dose at $d_{\text{max}}$ for a given applicator to that of the reference applicator at the same energy.^(20)^

(1) $$Output\;Factor(E,r,d_{\text{max}})=\frac{D_{w}(E,r,d_{\text{max}})}{D_{w}(E,r_{ref},d_{\text{max}}-ref)}$$

where $D_{w}$ is the absorbed dose to water, $d_{max}$ is depth of maximum dose for given applicator, *r* is the size of given applicator, $r_{ref}$ is the size of the reference applicator, $d_{max-ref}$ is depth of maximum dose for the reference applicator, and *E* is the electron beam energy.

The reference applicator for LIAC was the flat base cylindrical applicator with the diameter of 10 cm and for Varian it was the $10\times 10\;{\text{cm}}^{2}$ square applicator. In order to obtain the output factors for flat applicators of LIAC accelerator at various energies, Advanced Markus ion chamber was employed.^(27)^ Chamber was connected to a UNIDOSE E digital electrometer (PTW) and bias voltage was set to 300 volts. Then, the effective point of measurement of chamber was placed at the $d_{\text{max}}$ of each energy/cylindrical applicator size combination and absorbed dose was measured based on recommendations of the IAEA TRS‐398 protocol.^(16)^ Then, the output factors were determined using Eq. (1).

To determine the output factors of beveled applicators, the PinPoint chamber was employed.^(27)^ The effective point of measurement of detector, 3.4 mm from chamber tip on chamber axis, was shifted downstream by 0.5 mm $\left(0.5\;r_{\text{cavity}}\right)$ from $d_{\text{max}}$ of each energy/beveled applicator size combination and output factors were determined following Eq. (1). The chamber voltage was set to 400 volts and signal from the chamber was read using the UNIDOSE E digital electrometer. It should be mentioned that the PinPoint chamber was cross‐calibrated with the Advanced Markus chamber, using the maximum electron energy of LIAC.

For each output factor measurement, irradiation was repeated three times.

It should be mentioned that the Laitano formalism^(18)^ was used to determine the recombination correction factor of the employed ion chambers (Table 1). To determine the quality conversion factor at the $d_{\text{max}}$, the formalism proposed by IAEA TRS‐398 protocol^(16)^ was employed.

As reported by Table 1, the recombination correction factors calculated by Laitano formalism are considerably lower than those of calculated by standard TVA method (IAEA TRS‐398). Therefore, employing the standard TVA method during absolute dosimetry of introperative electron beam can largely overestimate the measured absorbed dose.

For measuring the output factors of Varian beam at different energies, the effective point of measurement of Advanced Markus chamber was placed at the reference depth of each square applicator inside the water and absorbed dose was determined. Then, the absorbed dose at the $d_{\text{max}}$ was derived using the measured PDD data. Irradiation was repeated three times for each employed applicator.

Finally, the output factors for each energy were calculated employing Eq. (1).

**Table 1:** The values of $K_{\text{sat}}$ for Advanced Markus and PinPoint chambers at different LIAC electron beam energies. Measurements were performed using the 10 cm flat applicator

	*Advanced Markus*		*PinPoint*	
*Energy (MeV)*	$K_{sat}$ *(Laitano)*	$K_{sat}$ *(TRS‐398)*	$K_{sat}$ *(Laitano)*	$K_{sat}$ *(TRS‐398)*
6	1.002	1.010	1.001	1.007
8	1.002	1.007	1.002	1.013
10	1.004	1.019	1.003	1.021
12	1.005	1.024	1.004	1.032

## III. RESULTS & DISCUSSION

### A. Percentage depth dose

**Figure 1:**
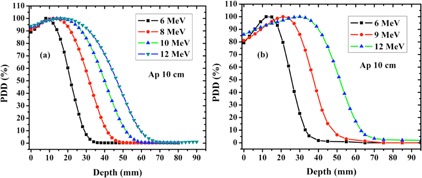
Depth dose distribution along the clinical axis for LIAC (a) and Varian beam (b) at different energies.

The PDDs of the electron beam from LIAC and Varian accelerators at different energies are shown in Fig. 1.

As expected, in both situations, with the energy increment, dose gradient at depth considerably decreases while surface dose increases. As it can be seen, the PDD curves derived from the LIAC fall more rapidly, which is a consequence of different beam collimation system used by this kind of radiotherapy accelerator.

As it previously mentioned, LIAC accelerator uses the cylindrical applicators and takes the hard docking mechanism for electron beam collimation. This kind of beam collimation causes a greater decrease in average of beam energy at the exit of IORT applicator due to the multiple scattering of electrons from applicator wall. This decrease in average energy will reduce the electron beam penetration inside the water and, as a consequence, LIAC PDDs fall more rapidly, compared to Varian PDDs.

PDD parameters related to the accelerators understudy are reported in Table 2.

As it appears in Table 2, $R_{100},R_{90}$, and $R_{P}$ for the LIAC beam are lower than those of the Varian beam. Also, for the same energies, the beam quality index, $R_{50}$, of the LIAC beam has lower values than that of the Varian accelerator, which could be attributed to the decrement in electron beam average energy at the exit of IORT applicator and the loss of side scatter equilibrium. Surface doses related to the LIAC beam are substantially higher than those of the Varian beam at all energies. This fact can be due to the both decrease in electron beam average energy, and smaller LIAC SSD in comparison to the Varian one. Furthermore, because no bending magnet is present in the LIAC structure, the low energy component of primary electron spectrum can't be removed from output electron beam at the exit of IORT applicator. Contribution of this low energy component causes a more increase in the surface dose.

**Table 2:** Electron beam parameters calculated from measured PDDs

*Varian 2100C/D*							*LIAC (12 MeV Model)*					
*Energy (MeV)*	$R_{100}$ *(mm)*	$R_{90}$ *(mm)*	$R_{P}$ (mm)	$R_{50}$ *(mm)*	$D_{s}$ (%)	*X‐ray Background absorbed dose (%)*	$R_{100}$ *(mm)*	$R_{90}$ *(mm)*	$R_{P}$ *(mm)*	$R_{50}$ *(mm)*	$D_{s}$ (%)	*X‐ray background absorbed dose (%)*
6	14.5	18.8	32.5	24.5	78	0.8	8.2	14	28.6	21.3	89	0.2
8	–	–	–	–	–	–	12.1	21.6	41.7	31.6	91	0.4
9	21.2	28.8	46	37.4	81	1.3	–	–	–	–	–	–
10	–	–	–	–	–	–	15.7	27.3	52.2	40	93	0.5
12	29.9	40.2	62.3	51.5	85.7	2	16	31.6	61.1	46.7	94	1

$R_{100},R_{90},R_{50}$ and $R_{P}=$ depths of maximum dose, 90% dose, 50% dose and practical range, respectively; $D_{s}=$ percentage depth dose at surface.

The PDD parameters measured in this work were in accordance with the data reported by Iaccarino et al.^(27)^ The mean difference between our results and mentioned study was equal to 4.5%.

For all energies, the photon contamination of the LIAC electron beam lies significantly lower than that of the Varian beam. The major sources of the X‐ray contamination are the bending magnet which is utilized to change the electron beam direction and collimation system including collimator jaws. Unlike conventional accelerators, neither X‐ray adjustable jaw nor bending magnet is used in the LIAC, and therefore the photon contamination stays in lower level, at all energies.

**Figure 2:**
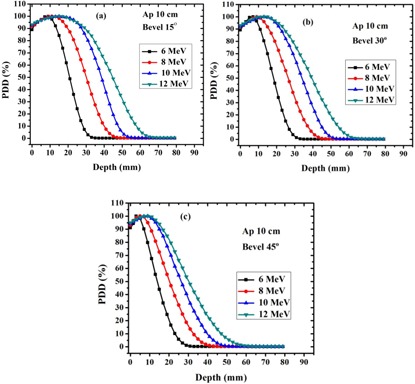
Depth dose distribution along the clinical axis for 15° (a), 30° (b), and 45° (c) beveled applicators at different energies of LIAC electron beam.

Measured PDDs along clinical axis of 10 cm beveled applicator at different combinations of energy/bevel angle are shown in Fig. 2.

As it can be seen, the PDDs of beveled applicators decrease more rapidly than those of flat applicators. Furthermore, the decrease in depth of penetration is more evident when the bevel angle increases.

PDD parameters related to the different bevel angles at different energies are reported in Table 3.

Except the surface dose, PDD parameters along the clinical axis of the beveled applicators are lower than those of the flat applicators at corresponding energies. This decrease is more evident when the bevel angle increases. With increasing the bevel angle, depth of maximum dose shifts to the shallower depth. Furthermore, employing the beveled applicator increases the surface dose up to 2.2% due to the oblique incidence of electron beam.

**Table 3:** PDD parameters of different beveled applicators with 10 cm diameter

*Energy (MeV)/Bevel Angle (degree)*	$R_{100}$ *(mm)*	$R_{90}$ *(mm)*	$R_{50}$ *(mm)*	$R_{P}$ *(mm)*	$D_{s}$ (%)
6/15	8	13.5	20.8	28.5	89
8/15	11.1	19.8	30.1	40.6	91.8
10/15	14.9	27.4	38.5	48.9	92.6
12/15	15.2	29.5	44.7	59.7	93.2
6/30	6.6	10.9	18.3	26.7	89.1
8/30	9	15.7	26.4	37.8	92
10/30	13	22.1	34	46.1	92
12/30	13.2	24.1	39.4	54.9	93
6/45	3	7	13.9	22.7	91
8/45	5.2	10.6	20.3	32.4	93
10/45	8.6	15	26.3	40.3	94.2
12/45	8.8	16.7	30.3	46.5	95

### B. Transverse dose profile

The TDPs for different energies of LIAC and Varian beam are shown in 3, 4, respectively. TDP parameters of each accelerator at various energies are reported in Table 4.

As it can be seen from Table 4, the symmetry and flatness of LIAC beam profiles are more desirable in comparison to those of the Varian. This difference can be explained by the differences in SSD, scattering foil design, and beam collimation system of the accelerators understudy. Furthermore, the left and right penumbras (the distance between isodose levels of 20% and 80% at both sides of the transverse dose profile) are considerably lower in the case of LIAC beam. This fact can be attributed to the type of electron collimation by this kind of accelerator and confining the electron beam to interior space of IORT applicator.

**Figure 3:**
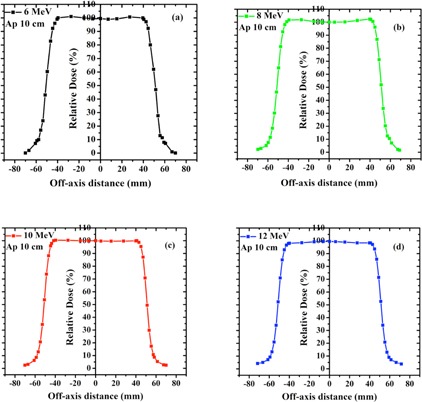
Measured TDPs at $d_{\text{max}}$ for different electron beam energies, 6 (a), 8 (b), 10 (c), and 12 MeV (d), produced by LIAC accelerator. Measurements were performed using flat applicator of 10 cm diameter.

**Figure 4:**
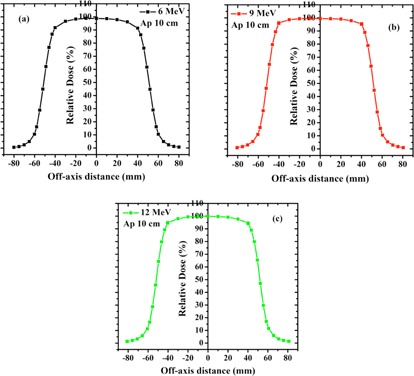
Measured TDPs at $d_{\text{max}}$ for 6 (a), 9 (b), and 12 MeV (c) electron beam produced by Varian accelerator. Measurements were carried out using the $10\times 10\;{\text{cm}}^{2}$ square applicator.

**Table 4:** Electron beam parameters calculated from measured TDPs

	*Varian 2100C/D*				*LIAC (12 MeV Model)*			
*Energy (MeV)*	*Left Penumbra (mm)*	*Right Penumbra (mm)*	*Flatness (%)*	*Symmetry (%)*	*Left Penumbra (mm)*	*Right Penumbra (mm)*	*Flatness (%)*	*Symmetry (%)*
6	11.3	11.5	2.3	0.5	8.3	8	1.2	1.4
8	–	–	–	–	7.7	7.2	1.3	0.7
9	10.7	10.6	1.4	0.7	–	–	–	–
10	–	–	–	–	7	6.7	0.7	0.8
12	10.5	10.6	2.1	0.6	7	7	0.9	0.4

### C. Output factor

**Figure 5:**
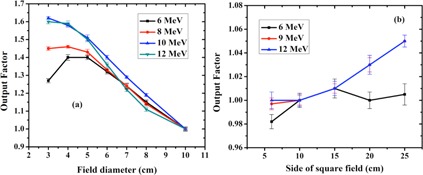
Variations of the output factor as a function of field size at different energies for LIAC (a) and Varian (b) electron beam.

Figure 5 illustrates the output factor for the electron beam produced by the LIAC and the Varian accelerators at different energies and field sizes. It should be mentioned that the LIAC and Varian output factors were measured at the SDD of 71.3 cm and 100 cm, respectively.

The results of the LIAC output factors were in agreement with the results reported by Iaccarino et al.^(27)^ The mean difference between the results of our study and those of the Iaccarino study for different combinations of energy/flat applicator size was equal to 2.7%.

As Fig. 5 depicts, with increasing the field size, the output factor of the Varian accelerator enhances which can be attributed to the increased contribution of scattered electrons in the radiation field. On the other hand, for the LIAC accelerator, cylindrical applicators with larger diameters experience a noticeable decrease in output factor. This fact is well justified by the Monte Carlo results reported by Pimpinella et al.^(20)^ The results of the Pimpinella study showed that the electron fluence at the exit of IORT applicator increases as the applicator diameter decreases. This increase in energy fluence causes a larger absorbed dose. Therefore, based on Eq. (1), the smaller applicators would have larger output factors.

Furthermore, the range of output factor variations with changing the field size is significantly higher in the case of LIAC accelerator, as it can be seen from Fig. 5.

Output factors measured for various combinations of applicator size/bevel angle at different energies of LIAC electron beam are reported in Table 5. As it can be seen from Table 5, output factor variations of beveled applicators with changing the field size are similar to those reported for flat applicators.

The mean difference between our results and those reported by Iaccarino et *al*.^(27)^ for beveled applicators was equal to1.5%.

**Table 5:** LIAC output factors for different combinations of energy/applicator size of beveled applicators

*Energy (MeV)/Bevel Angle (degree)*	*Applicator Diameter (cm)*						
	*3*	*4*	*5*	*6*	*7*	*8*	*10*
6/15	1.26	1.39	1.39	1.32	1.23	1.15	0.99
8/15	1.43	1.49	1.46	1.37	1.26	1.17	1.00
10/15	1.62	1.59	1.53	1.42	1.30	1.20	1.01
12/15	1.60	1.59	1.50	1.39	1.24	1.14	1.00
6/30	1.28	1.40	1.41	1.33	1.24	1.16	1.00
8/30	1.46	1.51	1.49	1.39	1.28	1.19	1.01
10/30	1.64	1.61	1.56	1.46	1.34	1.23	1.04
12/30	1.64	1.61	1.55	1.36	1.30	1.19	1.04
6/45	1.33	1.47	1.48	1.39	1.30	1.21	1.05
8/45	1.51	1.58	1.56	1.46	1.35	1.24	1.06
10/45	1.68	1.66	1.63	1.52	1.39	1.27	1.08
12/45	1.66	1.68	1.58	1.47	1.35	1.24	1.08

## IV. CONCLUSIONS

In this study, the dosimetric characteristics of the electron beam produced by LIAC, a mobile dedicated intraoperative radiotherapy accelerator, and Varian 2100C/D, a conventional radiotherapy accelerator, were evaluated and compared. Furthermore, the dosimetric characteristics of beveled IORT applicators along clinical axis were also reported.

The results of this work showed that the dosimetric characteristics of intraoperative electron beam are substantially different from those of conventional clinical electron beam. Surface dose at all energies is higher in the case of LIAC beam. Regarding to the fact that the surface (proximal end of tumor bed) is considered as a part of treatment target in IORT, increasing the surface dose is a preferred advantage. Furthermore, PDD of LIAC beam descend more rapidly, if compared to the Varian PDDs, which as a consequence, the underlying healthy tissues and organs at risk receive lower doses. But $R_{90}$ (the depth at which the IORT dose is prescribed^(28)^) for all energies of LIAC beam, is lower than Varian beam, which introduces a limitation on the suitable coverage of targets with the depths more than 3 cm.

Dosimetric characteristics of the beveled IORT applicators were substantially different from those of the flat ones. PDD parameters along clinical axis of beveled applicators were lower than those of the flat ones. Therefore, the therapeutic range of intraoperative electron beam along clinical axis decreases with employing the beveled applicators. On the other hand, due to the oblique incidence of electron beam, the surface dose increases using beveled IORT applicators.

TDP comparisons also cleared that the symmetry and flatness of LIAC electron fields are more favorable at all energies. According to this result, the uniformity of dose distribution over the target volume is improved by employing this mobile dedicated radiotherapy accelerator. Furthermore, due to the smaller penumbra region of the LIAC electron fields, surrounding healthy tissues will also receive lower doses.

Comparison of the output factors at different energies showed that, contrary to the Varian accelerator, output factor of the LIAC beam substantially increases with a decrease in the size of the applicator. Similar to the flat applicators, beveled IORT applicators with smaller diameter have larger output factor.

The results of this comparison showed that the dosimetric characteristics of the LIAC electron beam make it a useful tool for intraoperative radiotherapy purposes where the doses of clinical target volume and surrounding normal tissues are of main concern.
